# Intraocular Temperature at Different Sites in Eye Measured at the Beginning of Vitreous Surgery

**DOI:** 10.3390/jcm10153412

**Published:** 2021-07-31

**Authors:** Kei Shinoda, Kazuma Yagura, Soiti Matsumoto, Gaku Terauchi, Atsushi Mizota, Yozo Miyake

**Affiliations:** 1 Department of Ophthalmology, Teikyo University School of Medicine, 2-11-1, Itabashi-ku, Tokyo 173-8605, Japan; yagpiero@yahoo.co.jp (K.Y.); soiti@icloud.com (S.M.); jakkaruniomakase22@yahoo.co.jp (G.T.); mizota-a@med.teikyo-u.ac.jp (A.M.); 2 Department of Ophthalmology, Saitama Medical University Faculty of Medicine, 38 Morohongo, Moroyama-machi, Iruma-gun, Saitama 350-0495, Japan; 3 Matsumoto Eye Clinic, 50 Takagaki, Awacho, Awa-shi 771-1705, Tokushima, Japan; 4 Kobe City Eye Hospital, 2-1-8, Minamimachi, Minatojima, Chuo-ku, Kobe 650-0047, Hyogo, Japan; ymiyake@aichi-med-u.ac.jp

**Keywords:** vitreous surgery, intraocular temperature, silicone oil, avitreous

## Abstract

The temperature of the vitreous has been reported to vary during cataract and vitreous surgery. We measured intraocular temperature at four intraocular sites; the anterior chamber (AC), just behind the crystalline lens, mid-vitreous, and just anterior to the optic disc (OD) at the beginning of vitrectomy with a thermoprobe in 48 eyes. The temperatures were compared in three groups; eyes that underwent vitrectomy for the first time (Group V, *n* = 30), eyes that had previous vitrectomy and the vitreous cavity had been filled with balanced salt solution (BSS; Group A, *n* = 12), and eyes that had previous vitrectomy and the vitreous cavity was filled with silicone oil (Group S, *n* = 6). There was a gradient in the temperature in all groups, i.e., it was lowest in the AC, and it increased at points closer to the retina. The intraocular temperature was significantly correlated with the type of fluid in the vitreous cavity. The mean intraocular temperatures were not significantly different in Groups V and A, but they were significantly higher in Group S. Clinicians should be aware of the differences in the temperature at the different intraocular sites because the temperatures may affect the physiology of the retina and the recovery process.

## 1. Introduction

Vitreous surgery was first performed by Machemer and his colleagues in 1971 [1], and many studies have been performed on the effectiveness of new techniques and instruments in improving the outcomes [2]. There have also been studies that showed that the temperature of the vitreous humor is significantly lower than the body temperature [3]. In addition, it has been shown that the temperature of the vitreous during phacoemulsification [3,4] and also during different procedures such as core vitrectomy, endolaser photocoagulation, and membrane peeling of vitrectomy [4]. The baseline vitreous temperature is significantly lower at the end of vitrectomy [4,5,6]. However, how long the lower temperature lasts has not been determined.

The purpose of this study was to determine the temperature at 4 intraocular sites: in the anterior chamber (AC), just behind the crystalline lens, the mid-vitreous, and just anterior to the optic disc (OD) at the beginning of the vitreous surgery. To accomplish this, we used a thermoprobe to measure the temperature. These measurements were made in eyes that had not undergone vitrectomy, eyes in which the eyes had undergone vitrectomy and the vitreous cavity was filled with balanced salt solution (BSS Plus, Alcon Inc, Fort Worth, TX, USA) or silicone oil (SO). We also determined the correlations of the temperature with different clinical factors.

## 2. Patients and Methods

### 2.1. Patients

The participants were 119 patients scheduled to undergo vitrectomy as part of their treatment and had given consent for the operation and temperature measurements. The vitrectomies and temperature measurements were performed between November 2013 to July 2016 at the Teikyo University Hospital in Tokyo, Japan.

Temperature measurement was performed in 119 eyes at the beginning of surgery, but in 71 eyes, measurements at least one of four sites were missing because it was not strictly decided to measure the temperature of four sites. In 9 of 71 eyes, the temperature measurement was done only for midvitreous. In the other 62 eyes, at least the temperature at AC was not measured. The 62 eyes underwent vitrectomy alone and the measurement of AC was intentionally avoided because it required otherwise unnecessary paracentesis. In 19 of 48 eyes where temperature measurements at all four sites were done, AC temperature measurement was performed along with any manipulation in AC such as SO bubble removal, synechiolysis, or pupillary stretch. Finally, the measurements of all four sites were available only in the 48 eyes. The clinical characteristics of the patients are shown in Table 1. Thirty-four of the patients were men and 14 were women, and the average age of all was 60.0 ± 11.5 (±SD) years with a range from 35 to 82 years. The eyes were classified into three groups; eyes that underwent vitrectomy for the first time (Group V, *n* = 30), eyes that had undergone vitrectomy and their vitreous cavity was filled with balanced salt solution (BSS Plus, Alcon Inc, Fort Worth, TX, USA) at the end of vitrectomy (Group A, *n* = 12), and eyes that had undergone vitrectomy and their vitreous cavity was filled with SO (SILIKON^TM^ 1000, polydimethylsiloxane, Alcon Laboratories, Inc, Group S, *n* = 6). The averaged interval from the initial surgery was 248.5 ± 368.3 (±SD) (range 4–1297) days and 225.3 ± 114.9 (112–471) in Group V and Group S, respectively. The averaged interval from the preceding surgery was 195.2 ± 354.0 (±SD) (4–1297) days and 147.3 ± 47.1 (102–224) in Group V and Group S, respectively. The indications for the vitrectomy are listed in Table 1.

The procedures used in this study were approved by the Institutional Review Board of the Teikyo University (ID:10-033-2), and a written informed consent for the temperature measurements and surgical procedure was obtained from all of the participants.

### 2.2. Temperature Measurements

Temperature measurements were made with a 26-gauge flexible wire thermoprobe (Needle Microprobe MT-26/6, Physitemp Instruments LLC, Clifton, NJ, USA). The microprobe was connected to an analogue digital converter (PL3508 PowerLab 8/35, AD Instruments, Sydney, Australia), and data analyses were performed by the LabChart Pro software (AD Instruments, Sydney, Australia). The thermoprobe is accurate to within 0.1 °C and equilibrates with the surrounding fluid within 2 to 3 s. The temperature sensor is located at the tip of the probe. The thermoprobe was sterilized in ethylene oxide gas before use.

**Figure 1:**
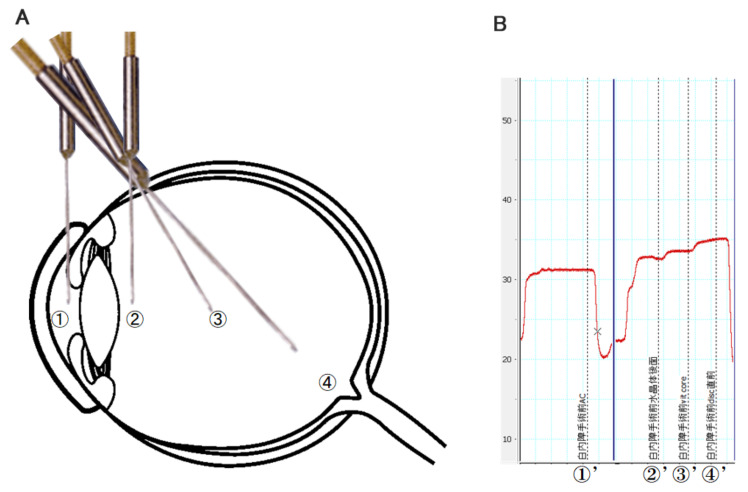
Schematic diagrams of the different intraocular sites for temperature measurements. (**A**) Location of intraocular temperature measurement using a thermoprobe. The temperature measurements were made by the sensor at the tip of the thermoprobe placed in the anterior chamber (1), behind the lens (2), mid-vitreous (3), and just above the optic disc (4). The thermoprobe was placed while viewing through an operating microscope. (**B**) Measurement of intraocular temperature at different locations at the beginning of the surgery. The tip of the thermoprobe was moved to measure the temperature of each site. The drop in the curve (X) of the temperature indicates that it has dropped to the room temperature because the tip of the thermometer was taken out of the eye. (1) Anterior chamber at the beginning of surgery; (2) behind lens at the beginning of surgery; (3) mid-vitreous at the beginning of surgery; (4) just anterior to the optic disc at the beginning of surgery.

All surgeries were performed with the CONSTELLATION^®^ Vision System (Alcon, Fort Worth, TX, USA) with 25-gauge instruments, and the surgeries were performed under sub-Tenon anesthesia. After prepping and draping in a sterile fashion, valved trocars were placed at the conventional sites for pars plana, and standard 25-gauge vitrectomy was performed. The temperature measurements were made just after the sclerotomy sites were made and before opening the infusion line. In phacovitrectomy (*n* = 29), temperature measurement was done just before cataract surgery and in vitrectomy alone, temperature measurement was done just before vitrectomy (*n* = 19), therefore temperature measurement was performed at the beginning of surgery in all 48 eyes so that any surgical procedures except placing trocar did not affect intraocular temperature. The measurements were made in the AC, just behind the crystalline lens, mid-vitreous, and just anterior to the OD. The room temperature was set at 25 °C, and the temperature of the patient’s skin was recorded. The thermoprobe was removed from the sterile package and connected to the digital thermometer. The clamped infusion line was connected but not opened, and the tip of the thermoprobe was inserted into the AC through a preplaced corneoscleral side port. Immediately after the temperature in the AC was measured, the thermoprobe was withdrawn and inserted into the vitreous cavity through the trocar. The location of the tip of the thermoprobe was controlled under view of a binocular indirect OphthalmoMicroscope (OCULUS Optikgeräte GmbH, Wetzlar, Germany), a wide-angle observation system for vitreous surgery. The intravitreal temperature measurements were taken by placing the tip of the thermoprobe just behind the crystalline lens or implanted intraocular lens, the mid-vitreous cavity, and the immediately anterior to the optic disc (Figure 1A). The total time required to record these measurements was approximately 1 min (Figure 1B). The thermoprobe was removed, and a standard vitrectomy was performed.

To minimize variability in the location of temperature measurements, the surgeon and surgical assistant localized the tip of the thermoprobe while viewing the microprobe through the operating microscope, and another assistant help guide the tip of the thermoprobe by examining a personal computer where the temperature change was displayed in real time.

### 2.3. Statistical Analyses

Comparisons of the temperature at the four different sites was done using analysis of variance followed by a post hoc test (Tukey–Kramer test). The significance of the differences in the temperatures among the three groups was determined by analysis of variance followed by post hoc test (Tukey–Kramer test). Multiple regression analysis was performed to determine the clinical factors that were significantly correlated with the intraocular temperature at each site. The explanatory variables were the age, sex, axial length, body temperature, lens status, and type of materials in the vitreous cavity. The objective variable was the temperature at each site. The lens status was crystalline lens or IOL and the vitreous filling material was intact vitreous (Group V), BSS (Group A), and SO (Group S). The JMP version 10.1 software (SAS Institute Inc., Cary, NC, USA) and Stata software version 15 (StataCorp LP, College Station, TX, USA) were used for the statistical analyses. A *p* < 0.05 was taken to be statistically significant.

## 3. Results

**Figure 2:**
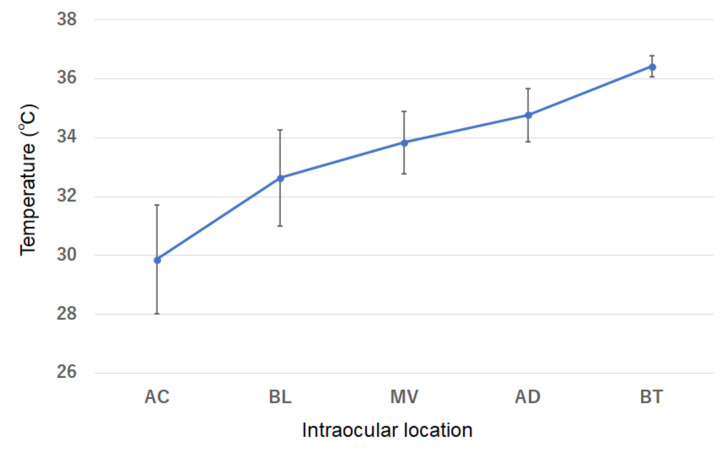
Distribution of the intraocular temperature at the beginning of vitrectomy in all eyes. The temperature was lower at the more anterior part of the eye. AC, anterior chamber, BL, behind lens; MV, mid-vitreous; OD; just anterior to the optic disc.

The intraocular temperature was 29.9 ± 1.8 °C in the AC, 32.6 ± 1.6 °C posterior to the lens, 33.8 ± 1.1 °C in the mid-vitreous, and 34.8 ± 0.9 °C just anterior to the OD. Thus, the temperature was lowest in the anterior part of the eye and gradually increased towards the posterior of the eye (Figure 2; Table 2). Interestingly, the temperature just anterior to the OD was lower than the body temperature.

**Figure 3:**
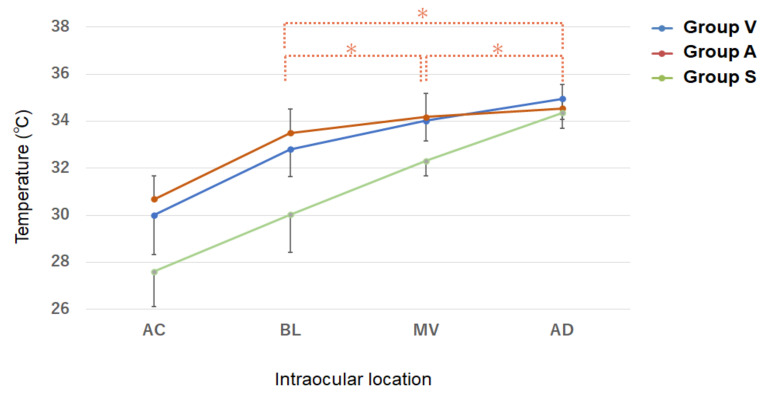
Distribution of the intraocular temperature at the beginning of vitrectomy in each group.

The temperatures in the three group are shown in Figure 3. In all three groups, the temperature was lowest in the AC and then increased gradually more posteriorly (Table 3). The gradient of change was similar in Groups V and A, but it was different in Group S. The temperature had a relatively large change from the AC to the back of the lens, and thereafter it increased linearly posteriorly in Groups V and A, whereas in Group S, the temperature gradient was linear without a gap.

The intraocular temperatures in eyes that were filled with different materials are shown. There was an increasing temperature gradient from anterior to posterior. Significant differences were observed in all comparisons between two sites except BL vs. MV, BL vs. OD, and MV vs. OD in Group A (*), suggesting that the intravitreal temperature change is minimal in Group A, Group V, eyes that underwent vitrectomy for the first time; Group A, eyes that had undergone vitrectomy and their vitreous cavity was filled with balanced salt solution; Group S, eyes that had undergone vitrectomy and their vitreous cavity was filled with silicone oil. AC, anterior chamber; BL, behind the lens; MV, mid-vitreous; OD, just anterior to the optic disc.

The temperature in the AC was 30.0 ± 1.7 °C in Group V, 30.7 ± 1.7 °C in Group A and 27.6 ± 1.5 °C in Group S The temperature was significantly lower in Group S than that in Groups V and A (Table 3). The temperature at the back of the lens was 32.8 ± 1.2 °C in Group V, 33.5 ± 1.5 °C in Group A and 30.0 ± 1.6 °C in Group S The temperature was significantly lower in Group S compared to that in the other groups (Table 3). The temperature at the mid-vitreous was 34.0 ± 0.9 °C in Group V, 34.2 ± 1.1 °C in Group A and 32.3 ± 0.6 °C in Group S The temperature was significantly lower in Group S than that in the other groups (Table 3). The temperature just anterior to the OD was 35.0 ± 0.9 °C in Group V, 34.6 ± 1.1 °C in Group A and 34.4 ± 0.7 °C in Group S. No significant differences were observed in the temperature among the three groups.

A significant difference was observed for all comparisons between two sites except between that posterior to the lens vs. mid-vitreous, between that posterior to the lens vs. anterior to the OD, and between that at the mid-vitreous and anterior to the OD in Group A (Table 2). These findings suggest that the intravitreal temperature gradient is minimal in an eye with BSS in the vitreous cavity.

Multiple regression analysis showed that the clinical factors that which were significantly correlated with the temperature behind lens (BL) was the axial length and vitreous filling material (Table 4). The factors that were significantly correlated with the mid-vitreous temperature were the sex and axial length, i.e., the longer the axial length, the higher the temperature was behind lens and at the mid-vitreous (Table 4). The temperature at the mid-vitreous was significantly higher in women than in men.

## 4. Discussion

Several studies have used infrared thermography to determine that the ocular surface temperature was different in normal subjects and in diseased eyes [7,8,9,10]. Other studies have shown that the ocular surface temperature decreases with age with a reduction of 0.010°C to 0.023°C/year. This was especially true for middle-aged and older individuals [7,8]. The difference in ocular surface temperature in phakic and pseudophakic patients was not significant in patients with cataracts, with clear crystalline lenses, and with pseudophakia [9]. The ocular surface temperature is higher in eyes with unilateral anterior uveitis compared to that of the contralateral eye [10]. However, little information is available on the intraocular temperatures [4,5,6].

It was recently reported that the vitreous humor is significantly hypothermic compared to the sublingual temperature [3]. It was also reported that vitreous temperature fluctuates during phacoemulsification [3,4] and during several procedures performed during vitrecomy [4,5,6]. Landers et al. [5] compared the intraocular temperature at three times; after preparation of the sclerotomy sites but before opening the infusion line, at end of active vitrectomy with an open infusion line, and at five minutes after closing of the infusion line and the sclerotomy sites. The mean mid-vitreous temperature was 33.9 °C before, 24.9 °C during and 30.6 °C immediately after vitrectomy. These differences were statistically significant. Thus, the vitreous temperature was significantly higher five minutes after closing the sclerotomies but was still lower than the baseline temperature. Unfortunately, the authors did not state when the temperature returned to the normal temperature.

Our study showed four main findings. First, the intraocular temperature was lower than the body temperature which is in good accordance with the earlier report by Landers et al. [5]. Our findings indicate that the hypothermia does not last long after surgery when the vitreous cavity was replaced by BSS but this was not the case when the vitreous cavity was filled with SO. In addition, the intraocular temperature differed depending on the fluid that filled vitreous cavity, and this has not been reported.

Second, the distribution of the intraocular temperature differed depending on the material in the vitreous cavity. Our results showed that the temperature just anterior to the optic disc was similar among the three groups; however, the in the vitreal space, especially the anterior part, was relative hyperthermic in SO-filled eye. This might be due to the difference in the specific heat property of the intravitreal materials. In addition, the metabolism and blood supply of the posterior segment may be one of the major factors that affects the intraocular temperature. The tissue temperatures of the retina, choroid, sclera, and bulbar conjunctiva were reported to be lower following a decrease in the choroidal blood flow [11]. It has been reported that patients with decreased ocular circulation such as those with carotid artery stenosis can have a reduced ocular surface temperature [12,13]. Because the SO has a higher specific heat index compared to BSS and natural vitreous, it is expected that SO-filled eye would be less susceptible to the effects of temperatures outside the body. The current results, however, were different.

Another possibility for the variations in the intraocular temperatures might be the effects of convection heat transfer. The differences might be due to the coefficient of viscosity of the materials. The temperature measurements were done when the patients were in a supine position for at least 10 min during the preparation for the surgery. Therefore, a warming effect of the choroidal circulation on the anterior segment of the eye may differ depending on the intraocular materials. The lower viscosity of the vitreous fluid and formed vitreous compared to SO may be associated with the higher intraocular temperature while in the supine position. This is important considering the association of the temperature to the intraocular oxygen pressure (pO_2_). The oxygen solubility decreases with higher temperatures thereby decreasing the pO_2_. The relative hypothermia in SO-filled eye may have be advantageous in this aspect.

Third, there was a temperature gradient so that the anterior segment was more hypothermic. This is not surprising considering that the surgical chamber was hypothermic and posterior segment of the eye was closer to the body trunk which is warmer. This intraocular temperature gradient may play some role in the transport and/or diffusion of relative high molecular substances, the so-called thermal diffusion. Metabolic activity, and the effectiveness of the intravitreally administrated biological agents such as anti-vascular endothelial growth factor antibody, may be affected by this. However, low viscosity media play a much higher role on the molecular transport [2] compared to the thermal diffusion in vitrectomized eyes. Our results show that the gradient was similar and the slope of the temperature gradient in the vitreous cavity was relatively slight in a cavity filled with BSS and non-vitrectomized eyes compared to that in SO-filled eyes. This may be associated with the specific heat index of the intravitreal material. Thermal convection may also play some role. Further investigations are needed to determine the physiological implications of the relative steep slope of the intravitreal temperature gradient in SO-filled eyes.

Fourth, the intraocular temperature may be associated with the axial length and sex. In eyes with longer axial lengths, the temperature just posterior to the lens and mid-vitreous would most likely be higher. Vitreous liquefactions in myopic eyes [14] may play some role. The temperature posterior to the lens and mid-vitreous was higher in Group A although it was not significantly higher. A plausible explanation might be a decrease in the choroidal circulation in eyes with longer axial lengths [15,16]; however, it may have an opposite effect.

The reason for the difference between the sexes in the temperature at the mid-vitreous was not determined. Sex differences in the location of the retinal vessels have been reported [17], and the heat retention effect of blood circulation may be different, but further research on the sex differences in the intraocular temperatures is needed.

It is known that even a slight decrease in vitreous temperature causes changes in the electroretinogram [18]. Because temperature has a considerable effect on retinal metabolism and function, vitreous surgery may affect retinal function not only during surgery but also after surgery [19,20,21], through the change of intraocular temperature profile in addition to the therapeutic effect of the retinal disease.

Our study has several limitations. The number of the vitrectomized eyes in Group A and Group S was relatively small. In addition, the pathology requiring the vitrectomy was different in each group. In eyes with retinal circulatory disorders, such as diabetic retinopathy, the choroidal circulation may be altered which may explain the differences in the intraocular temperature. Although several interesting observations were obtained by multiple regression analysis, further investigations are needed to confirm the results with a larger number of eyes. All the measurements were done when patients were in the supine position in the operating room where the temperature was set at 25 °C. Therefore, interpretation of the results should be carefully done. The results may be applicable to patients in the supine condition. The position of the patient during the procedures may influence the temperature gradient. It should also be considered that the environmental temperature and patients’ activity always changes.

## 5. Conclusions

In conclusion, we measured the intraocular temperature at four different sites at the beginning of vitreous surgery. Relative hyperthermia in the SO-filled eye, intraocular temperature gradient as hypothermia in the anterior part, and difference in the temperature distribution depending on the vitreous filling materials were observed. These findings may bring new aspect of insight into the physiological, pharmacological, and clinical consequences of vitrectomy. Temperature changes due to intravitreal substances may have some effect on the physiology of the vitreous.

## Figures and Tables

**Table 1:** Clinical characteristics in each group.

	All	Vitreous (Group V)	Avitreous (Group A)	SO (Group S)	*p* Value *
number	48	30	12	6	
male/female	34/14	21/9	8/4	5/1	
age(years) (mean±SD)	60.0 ± 11.5	62.1 ± 10.5	59.8 ± 13.0	50.3 ± 11.1	0.0758
body temperature (°C) (mean ± SD)	36.4 ± 0.4	36.4 ± 0.4	36.6 ± 0.4	36.3 ± 0.1	0.1715
lens status phakia/IOL	29/19	27/3	2/10	0/6	
Interval from initial surgery (days) (mean ± SD)			248.5 ± 368.3 (4–1297)	225.3 ± 114.9 (112–471)	0.8668
Interval from preceding surgery (days) (mean ± SD)			195.2 ± 354.0 (4–1297)	147.3 ± 47.1 (102–224)	0.7612
surgical indication					
PDR	15	10	5		
ERM	8	8			
RRD	19	7	6	6	
MH	2	2			
IOL dislocation	2	2			
VH	2	1	1		

SO; silicone oil, IOL; intraocular lens, PDR; proliferative diabetic retinopathy, ERM; epiretinal membrane, RRD; rhegmatogenous retinal detachment, MH; macular hole, VH; vitreous hemorrhage. * Comparison among three groups was made using ANOVA.

**Table 2:** Comparison of temperature between intraocular locations.

	ANOVA	AC vs. BL	AC vs. MV	AC vs. AD	BL vs. MV	BL vs. AD	MV vs. AD
Total	*<0.0001*	*<0.0001*	*<0.0001*	*<0.0001*	*0.0003*	*<0.0001*	*0.009*
Vitreous (Group V)	*<0.0001*	*<0.0001*	*<0.0001*	*<0.0001*	*0.0009*	*<0.0001*	*0.0162*
Avitreous (Group A)	*<0.0001*	*<0.0001*	*<0.0001*	*<0.0001*	0.6045	0.2432	0.9119
SO (Group S)	*<0.0001*	*0.0108*	*<0.0001*	*<0.0001*	*0.0159*	*<0.0001*	*0.0352*

AC; anterior chamber, BL; behind lens, MV; midvitreous, AD; above the optic disc, SO; silicon oil.

**Table 3:** Intraocular temperature in each group.

	AC	BL	MV	AD
temperature (°C)				
Total	29.9 ± 1.8	32.6 ± 1.6	33.8 ± 1.1	34.8 ± 0.9
Vitreous (Group V)	30.0 ± 1.7	32.8 ± 1.2	34.0 ± 0.9	35.0 ± 0.9
Avitreous (Group A)	30.7 ± 1.7	33.5 ± 1.5	34.2 ± 1.1	34.6 ± 1.1
SO (Group S)	27.6 ± 1.5	30.0 ± 1.6	32.3 ± 0.6	34.4 ± 0.7
*p* value				
ANOVA	*0.0027*	*<0.0001*	*0.0004*	0.2099
Vitreous vs. avitreous	0.4671	0.2868	0.855	0.3943
Vitreous vs. SO	*0.0082*	*<0.0001*	*0.0006*	0.3504
Avitreous vs. SO	*0.0022*	*<0.0001*	*0.0007*	0.9018

AC; anterior chamber, BL; behind lens, MV; midvitreous. AD; above the optic disc, SO; silicone oil.

**Table 4:** Clinical factors that influence on the intravitreal temperature at each site.

	AC		BL		MV		AD	
**ANOVA (*p* value)**	*0.0177*		*<0.0001*		*<0.0001*		0.0588	
	F value	*p* value	F value	*p* value	F value	*p* value	F value	*p* value
Age	0.642	0.4284	0.0676	0.7964	0.7096	0.4053	0.4517	0.5059
Sex *	0.4716	0.4968	0.1137	0.738	7.3879	*0.0101*	1.948	0.1716
Body temperature	0.6579	0.4228	1.674	0.2042	0.4947	0.4865	0.055	0.816
Lens status **	0.5969	0.4449	0.0005	0.9814	0.0057	0.9401	0.5111	0.4794
Axial length	1.6716	0.2045	5.1279	*0.0298*	11.7673	*0.0016*	3.0962	0.0872
Vitreous filling material ***	3.062	0.0595	6.707	*0.0034*	1.3724	0.2668	0.5847	0.5626

AC, anterior chamber; BL, behind lens; MV, midvitreous; AD, above the optic disc; SO, silicone oil. For the multiple regression analysis, sex, lens status, and vitreous filling material was converted to the order scale. * female; 0 male; 1, ** crystalline lense; 0, intraocular lens; 1, *** avitreous; 0, vitreous; 1, silicone oil; 2.

## Data Availability

The data are not publicly available due to personal information protection.

## References

[1] Machemer R., Buettner H., Norton E.W., Parel J.M. (1971). Vitrectomy: A pars plana approach. Trans. Am. Acad. Ophthalmol. Otolaryngol..

[2] Stefánsson E. (2009). Physiology of vitreous surgery. Graefes. Arch. Clin. Exp. Ophthalmol..

[3] Salcedo-Villanueva G., Kon-Jara V., Harasawa M., Cervantes-Coste G., Ochoa-Contreras D., Morales-Cantón V., Guerrero-Naranjo J.L., Quiroz-Mercado H., Landers M.B. (2015). Vitreous humor thermodynamics during phacoemulsification. Int. Ophthalmol..

[4] Iguchi Y., Asami T., Ueno S., Ushida H., Maruko R., Oiwa K., Terasaki H. (2014). Changes in vitreous temperature during intravitreal surgery. Investig. Ophthalmol. Vis. Sci..

[5] Landers M.B., Watson J.S., Ulrich J.N., Quiroz-Mercado H. (2012). Determination of retinal and vitreous temperature in vitrectomy. Retina.

[6] Romano M.R., Vallejo-Garcia J.L., Romano V., Angi M., Vinciguerra P., Costagliola C. (2013). Thermodynamics of vitreoretinal surgery. Curr. Eye Res..

[7] Alio J., Padron M. (1982). Influence of age on the temperature of the anterior segment of the eye. Measurements by infrared thermometry. Ophthalmic Res..

[8] Acharya U.R., Ng E.Y., Yee G.C., Hua T.J., Kagathi M. (2009). Analysis of normal human eye with different age groups using infrared images. J. Med. Syst..

[9] Sniegowski M., Erlanger M., Velez-Montoya R., Olson J.L. (2015). Difference in ocular surface temperature by infrared thermography in phakic and pseudophakic patients. Clin. Ophthalmol..

[10] Mapstone R. (1968). Determinants of corneal temperature. Br. J. Ophthalmol..

[11] Auker C.R., Parver L.M., Doyle T., Carpenter D.O. (1982). Choroidal blood flow. I. Ocular tissue temperature as a measure of flow. Arch. Ophthalmol..

[12] Galassi F., Giambene B., Corvi A., Falaschi G. (2007). Evaluation of ocular surface temperature and retrobulbar haemodynamics by infrared thermography and colour Doppler imaging in patients with glaucoma. Br. J. Ophthalmol..

[13] Morgan P.B., Smyth J.V., Tullo A.B., Efron N. (1999). Ocular temperature in carotid artery stenosis. Optom. Vis. Sci..

[14] Holekamp N.M., Harocopos G.J., Shui Y.B., Beebe D.C. (2008). Myopia and axial length contribute to vitreous liquefaction and nuclear cataract. Arch. Ophthalmol..

[15] Samra W.A., Pournaras C., Riva C., Emarah M. (2013). Choroidal hemodynamic in myopic patients with and without primary open-angle glaucoma. Acta Ophthalmol..

[16] Mori F., Konno S., Hikichi T., Yamaguchi Y., Ishiko S., Yoshida A. (2001). Factors affecting pulsatile ocular blood flow in normal subjects. Br. J. Ophthalmol..

[17] Noma S., Yamashita T., Asaoka R., Terasaki H., Yoshihara N., Kakiuchi N., Sakamoto T. (2020). Sex judgment using color fundus parameters in elementary school students. Graefes. Arch. Clin. Exp. Ophthalmol..

[18] Horiguchi M., Miyake Y. (1991). Effect of temperature on electroretinograph readings during closed vitrectomy in humans. Arch. Ophthalmol..

[19] Miyake Y., Yagasaki K., Horiguchi M. (1991). Electroretinographic monitoring of retinal function during eye surgery. Arch. Ophthalmol..

[20] Matsumoto C.S., Shinoda K., Terauchi G., Matsumoto H., Mizota A., Miyake Y. (2015). Assessment of Macular Function during Vitrectomy: New Approach Using Intraoperative Focal Macular Electroretinograms. PLoS ONE.

[21] Yagura K., Shinoda K., Matsumoto S., Terauchi G., Watanabe E., Matsumoto H., Akiyama G., Mizota A., Miyake Y. (2016). Intraoperative Electroretinograms before and after Core Vitrectomy. PLoS ONE.

